# Perturbation of the Dimer Interface of Triosephosphate Isomerase and its Effect on *Trypanosoma cruzi*

**DOI:** 10.1371/journal.pntd.0000001

**Published:** 2007-10-31

**Authors:** Vanesa Olivares-Illana, Adela Rodríguez-Romero, Ingeborg Becker, Miriam Berzunza, Juventino García, Ruy Pérez-Montfort, Nallely Cabrera, Francisco López-Calahorra, Marieta Tuena de Gómez-Puyou, Armando Gómez-Puyou

**Affiliations:** 1 Instituto de Fisiología Celular, Universidad Nacional Autónoma de México, Mexico City, Mexico; 2 Instituto de Química, Universidad Nacional Autónoma de México, Mexico City, Mexico; 3 Facultad de Medicina, Universidad Nacional Autónoma de México, Mexico City, Mexico; 4 Facultad de Química, Universidad Nacional Autónoma de México, Mexico City, Mexico; 5 Departamento de Química Orgánica, Universidad de Barcelona, Barcelona, Spain; University of Technology, Sydney, Australia

## Abstract

**Background:**

Chagas disease affects around 18 million people in the American continent. Unfortunately, there is no satisfactory treatment for the disease. The drugs currently used are not specific and exert serious toxic effects. Thus, there is an urgent need for drugs that are effective. Looking for molecules to eliminate the parasite, we have targeted a central enzyme of the glycolytic pathway: triosephosphate isomerase (TIM). The homodimeric enzyme is catalytically active only as a dimer. Because there are significant differences in the interface of the enzymes from the parasite and humans, we searched for small molecules that specifically disrupt contact between the two subunits of the enzyme from *Trypanosoma cruzi* but not those of TIM from *Homo sapiens* (HTIM), and tested if they kill the parasite.

**Methodology/Principal Findings:**

Dithiodianiline (DTDA) at nanomolar concentrations completely inactivates recombinant TIM of *T. cruzi* (TcTIM). It also inactivated HTIM, but at concentrations around 400 times higher. DTDA was also tested on four TcTIM mutants with each of its four cysteines replaced with either valine or alanine. The sensitivity of the mutants to DTDA was markedly similar to that of the wild type. The crystal structure of the TcTIM soaked in DTDA at 2.15 Å resolution, and the data on the mutants showed that inactivation resulted from alterations of the dimer interface. DTDA also prevented the growth of *Escherichia coli* cells transformed with TcTIM, had no effect on normal *E. coli*, and also killed *T. cruzi* epimastigotes in culture.

**Conclusions/Significance:**

By targeting on the dimer interface of oligomeric enzymes from parasites, it is possible to discover small molecules that selectively thwart the life of the parasite. Also, the conformational changes that DTDA induces in the dimer interface of the trypanosomal enzyme are unique and identify a region of the interface that could be targeted for drug discovery.

## Author Summary

Most of the enzymes of parasites have their counterpart in the host. Throughout evolution, the three-dimensional architecture of enzymes and their catalytic sites are highly conserved. Thus, identifying molecules that act exclusively on the active sites of the enzymes from parasites is a difficult task. However, it is documented that the majority of enzymes consist of various subunits, and that conservation in the interface of the subunits is lower than in the catalytic site. Indeed, we found that there are significant differences in the interface between the two subunits of triosephosphate isomerase from *Homo sapiens* and *Trypanosoma cruzi* (TcTIM), which causes Chagas disease in the American continent. In the search for agents that specifically inhibit TcTIM, we found that 2,2′-dithioaniline (DTDA) is far more effective in inactivating TcTIM than the human enzyme, and that its detrimental effect is due to perturbation of the dimer interface. Remarkably, DTDA prevented the growth of *Escherichia coli* cells that had TcTIM instead of their own TIM and killed *T. cruzi* epimastigotes in culture. Thus, this study highlights a new approach base of targeting molecular interfaces of dimers.

## Introduction

Triosephosphate isomerase (TIM) is a ubiquitous enzyme that catalyzes the interconversion between glyceraldehyde 3-phosphate and dihydroxyacetone phosphate. In most of the species the enzyme is formed by two identical monomers of approximately 250 amino acids. TIM belongs to the family of α-β barrels proteins, in which 8 central β strands are surrounded by 8 α helices; the strands and helices are joined by loops. It is one of the most thoroughly studied enzymes. Its kinetics are well established [1,2], the crystal structure of the enzyme from 15 different species is available, and significant advances have been made on the dynamics of the enzyme when it is in the resting state and during active catalysis [3–5].

A peculiarity of TIM is that only in its dimeric form the enzyme exhibits high catalytic rates, albeit each monomer has its own catalytic residues [6–8]. Along this line, it has been reported that deletion of some residues of loop3 in TIM from *Trypanosoma brucei* (TbTIM), which forms an important portion of the interface, yields a monomeric enzyme with drastically reduced catalytic activity [9,10]. Likewise, it has been shown that chemical perturbation by thiol reagents of the interfacial Cys15 of TbTIM, and that of TIMs from *T. cruzi* (TcTIM), *Leishmania mexicana* (LmTIM) [11], *Plasmodium falciparum* [12], and *Entamoeba histolytica* [13] induces drastic changes in the quaternary and tertiary structure of the respective dimers and abolition of catalytic activity.

The latter observations raised the question as to whether agents that interfere with protein-protein interactions in either permanent of transient oligomers, could be exploited for the discovery of molecules with pharmacological potential [14,15]. From the point of view of drug discovery for diseases that are caused by parasites, the possibility is particularly attractive. This is because in the course of evolution the catalytic site of enzymes has been largely conserved, whereas the amino acids that form the interfaces of oligomeric enzymes have undergone significant changes [16–18]. For example, TIMs from human and the aforementioned parasites have the same catalytic residues; in contrast, the identity of the approximately 32 interfacial residues of TIM from either of the parasites and human is approximately 52%, whereas the identity of the amino acid residues of the interface of the enzymes from *T. cruzi*, *T. brucei* and *L. mexicana* is approximately 82% [8,15]. Therefore, it is theoretically possible to find molecules that exhibit a high specificity for the interface of oligomeric enzymes from parasites. Based on the amino acid composition and structural differences of the interfaces of TIM, we have found agents that by acting on the dimer interface exhibit a high specificity for TIM from parasites [15].

In our attempts to find more effective low molecular weight compounds, we synthesized 3-(2-benzothiazolylthio)-1 propanethioaniline. We found that it induced a strong inhibition of enzyme activity. However, subsequent studies showed that the inhibition was not due to this compound, but to a contaminant that formed during its preparation. This proved to be 2,2′-dithiodianiline, (DTDA). Here we describe the action of DTDA on the function and structure of TcTIM. The compound is highly selective for TcTIM. We also found that DTDA inhibited the growth of *Escherichia coli* that had been transformed with TcTIM, and that in the low micromolar range, the compound also caused the death of *T. cruzi* epimastigotes. The crystal structure of the enzyme treated with DTDA complex showed that it induced important and rather unique alterations of the dimer interface.

## Materials and Methods

TcTIM [19] and TIMs from *T. brucei* [20], *L. mexicana* [21], and *Homo sapiens* [22] were expressed in *Escherichia coli* and purified as described in the indicated references. After purification, the enzymes dissolved in 100 mM triethanolamine, 10 mM EDTA and 1 mM dithiothreitol (pH 8) were precipitated with ammonium sulfate (75% saturation) and stored at 4°C. Before use, the enzymes were extensively dialyzed against 100 mM triethanolamine/10 mM EDTA (pH 7.4). Protein was determined from their absorbance at 280 nm [23]. The ε M^−1^ cm^−1^ was 36440 for TcTIM, and 33460 for TbTIM and LmTIM.

### Synthesis of 2,2′-dithiodianiline (DTDA)

**Figure 1:**
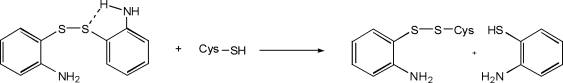
Reaction of DTDA with cysteine. The reaction of DTDA with the thiol group of Cys could be favored by the formation of an intramolecular hydrogen bond and the inductive effect of the amino group due to the higher electronegativity of the nitrogen atom with respect to the sulfur atom.

A solution of 1 g of 2-aminothiophenol in 10 ml dichloromethane was stirred at room temperature for 8 h to promote its oxidation. The residual material was washed with hexane and purified by silica gel chromatography with dichloromethane as eluent to give 0.97 g of DTDA. The product was characterized by mass spectrometry and NMR analysis. DTDA is spontaneously formed from 2-aminothiophenol and its reactivity is typical of disulfides. Its reaction with sulfides, such as cysteine, yields a mixed disulfide (Fig. 1). Indeed, as shown in the Results section, we found that Cys118 of wild type TcTIM reacts with DTDA forming a disulfide bond between Cys118 and thioaniline. The reaction would be analogous to the standard opening of disulfide bridges by reaction with an excess of β-mercaptoethanol or dithiothreitol.

In all the experiments, a solution of DTDA in dimethylsulfoxide (DMSO) was used. The final DMSO concentration in all experiments was 10% (v/v). It is noted that that at this concentration, DMSO did not affect the activity of the enzymes that were used.

### Assay of the action of DTDA on the various TIMs

The indicated TIMs were incubated at pH 7.4 at a concentration of 5 µg per ml of 100 mM triethanolamine, 10 mM EDTA, 10% dimethyl sulfoxide (v/v), and DTDA at the concentrations indicated in the Results section for 2 hours at 36°C. At this time, an aliquot was withdrawn for assay of activity.

### Activity

Activity was determined in the direction of glyceraldehyde 3-phosphate to dihydroxyacetone phosphate [11]. The decrease in absorbance at 340 nm was followed in a Hewlett Packard spectrophotometer at 25°C. The reaction mixture (1 ml) contained 100 mM triethanolamine, 10 mM EDTA, 0.2 mM NADH, 1 mM glyceraldehyde 3-phosphate, and 0.9 units α-glycerolphosphate dehydrogenase (pH 7.4). The reaction was started by the addition of TIM, usually 5 ng. The average specific activity of the various preparations of TcTIM used in this work was 2900±200 µmol/min/mg.

### Culture of *Escherichia coli*

*E. coli* JM103 cells were used as control. *E. coli* devoid of their endogenous TIM termed VR101 [10] were kindly provided by Dr. Gloria Saab-Rincón; these cells have a kanamicin resistant cassette. The latter cells were transformed with the plasmid pTrc99aTcTIM that had an ampicilin resistant cassette. The strains were grown at 37°C in solid Luria-Bertani medium that had been supplemented with 50 µg of kanamycin and 50 µg of ampicilin per ml. One colony was transferred to M9 medium that had 10% DMSO and 50 µg of each of the latter antibiotics per ml. Growth of the various cells was followed throughout time by measuring the absorbance of the culture at 600 nm.

### Culture of parasites

To study the effect of DTDA on the *T. cruzi*, 10^6^ epimastigotes of the strain ninoa were inoculated into RPMI 1640 media supplemented with 10% fetal bovine serum (Gibco, BRL, Rockville, Md); the media also had 10% DMSO (v/v); at this concentration DMSO did not exert a detrimental effect on the growth of *E. coli* cells, nor on the growth and survival of *T. cruzi* epimastigotes. Where indicated in the Results section, the media was supplemented with the indicated concentrations of DTDA. The number of cells was recorded at various times for as long as 72 hours.

### Soaking of TcTIM crystals with DTDA and data collection

We attempted to co-crystallize TcTIM with DTDA; we tried different concentrations of enzyme and DTDA, but all our attempts were unsuccessful. However, we succeeded in obtaining crystals of the complex by soaking crystals of TcTIM with DTDA. TcTIM was crystallized by the vapor diffusion hanging drop method. TcTIM, 2.5 µg in 5 µl of 25 mM triethanolamine (pH 8.0) was mixed with 5 µl of reservoir solution (0.1 M Na-Hepes, pH 7.5, 2% (v/v) PEG 400, and 2.0 M ammonium sulfate). Crystals appeared after two or three weeks. At this time, 1 µl of 10 mM DTDA was added to the drop (1 mM final concentration). After 48 hours the crystal was transferred to a cryoprotectant solution (30% (v/v) glycerol) and flash frozen. Diffraction data were collected at 113 K with a Rigaku X-ray rotating anode generator and an R-Axis IIC image plate detector. The data was processed and scaled with d*TREK [24].

### Structure determination and refinement

The 3D structure was solved using molecular replacement with the program MOLREP [25] and the coordinates of native TcTIM (PDB code 1TCD) as the search model. Refinement was carried out first with the program CNS [26], and manual adjustments of the model into electron density maps were done using QUANTA2000 (Accelrys). Five percent of the reflections were set aside for validation. The anisotropic motion of the subunits was described with the TLS parameters as implemented in REFMAC5 [27]. Both the molecular replacement and TLS refinement of the structure were done using the CCP4 program suite version 5.2.0005 [28]. A summary of the data-collection and refinement statistics is given in Table I. The coordinates of the structure have been deposited in the Protein Data Bank (PDB code 2OMA).

## Results

**Figure 2:**
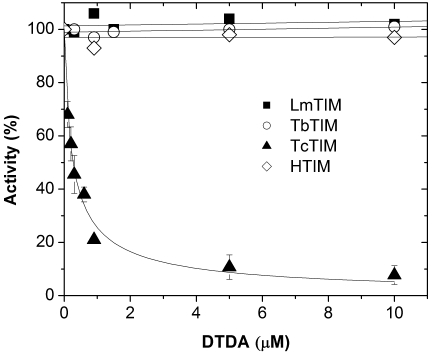
Effect of DTDA on the activity of TIMs from *T. cruzi, T. brucei, L. mexicana* and *Homo sapiens*. The enzymes were incubated at a concentration of 5 µg/ml in media that contained 100 mM triethanolamine, 10 mM EDTA, 10% DMSO (v/v) and the indicated concentrations of DTDA. After two hours of incubation at 36°C, aliquots were withdrawn for assay of activity. 100% corresponds to the activity of TcTIM, TbTIM, LmTIM, and human TIM at time zero; these activities were 2950, 3125, 4560, and 4900 µmol/min/mg, respectively.

As noted, we found that a contaminant that formed during the synthesis of 3-(-2-benzothiazolylthio)-1 propanethioaniline was a powerful inhibitor of the activity of TcTIM. The contaminant was identified as 2,2′-dithiodianiline (DTDA). The compound was subsequently synthesized, and the product characterized by mass spectrometry and NMR analysis. When the effect of DTDA was assessed in TIMs from various sources (Fig. 2), it was found that 260 nM induced 50% inhibition of the activity of TcTIM, and that 10 µM did not affect the activity of TbTIM, LmTIM and TIM from *H. sapiens* (HTIM). In connection to the inhibiting effect of DTDA on TcTIM, it is pointed out that the inhibition of activity was accompanied by aggregation of the enzyme, indicating that the compound induced drastic structural alterations. It is also noted, that at concentrations higher than those used in the experiment of Table 1, DTDA affected the activity of HTIM. Fifty percent inhibition of HTIM activity was achieved with 98 µM DTDA; thus, the selectivity for TcTIM in reference to HTIM is nearly 400-times. It is also noteworthy that TbTIM and LmTIM were completely insensitive to DTDA concentrations as high as 200 µM.

**Table 1:** Data Collection and Reduction Statistics for DTDA treated TcTIM.

Data collection and reduction	
Space group	P2_1_2_1_2_1_
Unit cell dimension (Å)	a = 43.21, b = 75.34, c = 146.43
Total number of reflections	82720
Unique reflections	23141
Resolution (Å)	2.15
Completeness (%)	91.0
R_merge_ (%)	9.3
Vm (Å^3.^Da^−1^)	2.3
Redundancy	3.4
*Refinement*	
Protein atoms/ligand atoms/water molecules	3850/92/268
Modified Cys residues with DTBA	2 (C118A and C118B)
Resolution range for refinement (Å)	41.45–2.15
Number of reflections (refinement)	23141 (1248 Rfree)
R/R_free_	0.197/0.250
RMS deviation (bond/angle/torsion)	0.022 Å/1.9°/18.5°

R = Σ|Fo-Fc|/ΣFo, summed over all hkl's

Since the three trypanosomatidal TIMs are very similar in amino acid sequence and three-dimensional structure [29–31], it was considered of interest to ascertain the mechanism through which DTDA affects the activity of TcTIM. In a first approach, we determined if the action of DTDA on TcTIM is related to the presence of the disulfide bond between the two aniline moieties. Accordingly, we synthesized and examined the action of 1,1′-(methylenebisthioaniline). In this compound the two aniline moieties are joined by –S-C-S- bonds. In contrast to DTDA which induces half-maximal inhibition at a concentration of 260 nM, 50 µM of 2,2′-(methylenebisthioaniline) was required to inhibit the activity of TcTIM by 50%. Clearly, the existence of the disulfide bond is central for optimal detrimental actions of DTDA.

Because disulfide compounds can induce the thioalkylation of free –SH groups, we assessed if the action of DTDA is related to the cysteine content of the trypanosomatid enzymes. The three enzymes have three common cysteines, those at positions 15, 41 and 127 (numbering system of TcTIM); TcTIM and LmTIM have an additional cysteine at position 118. Thus, in a first approach, we measured the effect of DTDA on a mutant of TcTIM in which its Cys118 was replaced by Val, the residue that exists in TbTIM. It was reasoned that if the deleterious action of DTDA on wild type TcTIM was due to perturbation of Cys118, the activity of the C118V mutant would be insensitive to DTDA. However, we found that the susceptibilities of the mutant and wild type enzymes to DTDA were almost identical (Table 2). This indicates that Cys118 of TcTIM does not play a central role in the inhibition of TcTIM by DTDA. These data are in consonance with the lack of effect of DTDA on LmTIM which has a cysteine in position 118. Moreover, we found that a mutant of TbTIM in which its Val118 was replaced by Cys was also insensitive to 200 µM DTDA (Table 2). Collectively, these data indicate that the detrimental action of DTDA on TcTIM does not depend on Cys118.

**Table 2:** Concentrations of DTDA that induces 50% inhibition in TcTIM and some of its mutants.

Enzyme	IC_50 (_µM)
**TcTIM wild**	0.258±0.035
**TcTIM C118V**	0.208±0.037
**TbTIM V118C**	NE
**LmTIM**	NE
**TcTIM C15A**	0.346±0.032
**TcTIM C40A** *	1.532±0.450
**TcTIM C127A**	0.057±0.0080

The effect of DTDA on the indicated enzymes was determined as in Figure 1, from the data the concentration that induced half-maximal inhibition was calculated. NE indicates that 200 µM DTDA had no effect.

* As noted in the text, relatively low DTDA concentrations inhibit the activity by approximately 50%; significantly higher concentrations were required for full inhibition. The data in the Table indicate the concentration at which half-maximal inhibition of the initial phase was achieved.

As noted the three-dimensional structure of TcTIM, TbTIM, and LmTIM are strikingly similar. Of particular interest is that in the three enzymes the side chain of Cys15 of each of the two monomers is surrounded by residues of loop3 of the adjoining subunits. It is also relevant that in the three enzymes, the geometrical arrangements of the residues of loop3 around Cys15 in TcTIM and TbTIM are markedly similar. Regardless of the similarities, the susceptibility of Cys15 of TcTIM to thiol reagents, such as dithionitrobenzoic acid and methylmethane thiosulfonate, is about two orders of magnitude higher than in TbTIM and LmTIM [11]. Apparently, this is related to the higher intrinsic flexibility of TcTIM [32]. Thus, we studied if the existence of Cys15 in TcTIM was instrumental in the action of DTDA. To this end, we determined the susceptibility of a C15A mutant of TcTIM to DTDA. The results showed that C15A TcTIM exhibited only a slightly lower sensitivity (Table 2). Therefore, the data on the TcTIM mutants indicate that the existence of cysteines, either at positions 15 or 118 is not necessary for the detrimental action of DTDA.

The effect of the compound was also studied in C127A and C40A TcTIM mutants. The results showed that the C127A mutant was more sensitive to DTDA than the wild type (Table 2). It has been previously shown that the C127A mutation decreases the stability of the enzyme [33]; thus, it is likely that the higher sensitivity to DTDA is related to its lower intrinsic stability. Nonetheless, the data with the C127A mutant indicate that Cys127 is not involved in the detrimental action of DTDA (Table 2). The effect of the compound was also assayed on the C40A mutant. Similarly to the wild type, the activity of this mutant was inhibited by about 50% by nM DTDA concentrations, however, it was also observed that much higher concentrations were needed to induce full inhibition of activity. The reasons for this biphasic response are not clear.

### X-ray structure of TcTIM soaked in DTDA

**Figure 3:**
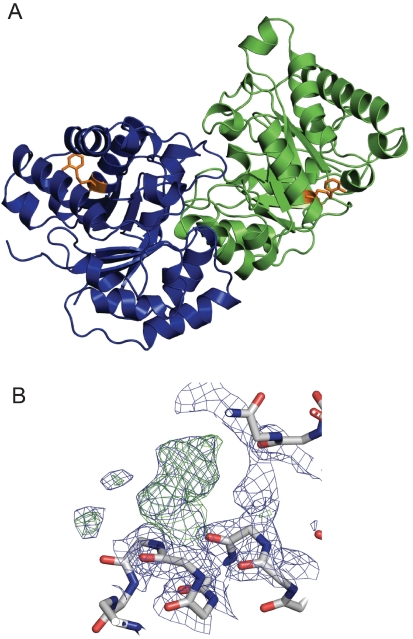
A. Overall structure of the TcTIM soaked in DTDA. B. Cysteine 118 is covalently bound to thiobenzylamine. Panel A shows that the general structure of the TcTIM dimer is not altered by DTDA; monomers A and B are shown in green and blue, respectively and thioaniline in orange. The figures were prepared with PyMOL. In panel B, the derivatized Cys118 of monomer B is shown. FoFc map (3 σ) after the first refinement using CNS annealing and B refinement; a clear positive density that corresponds to covalently bound thioaniline is observed. The same data were observed in monomer A (not shown).

To gain insight into the mechanism through which DTDA affects TcTIM, we attempted to co-crystallize the enzyme in complex with DTDA, however, our efforts were unsuccessful. On the other hand we were able to obtain crystals of the complex by soaking crystals of TcTIM with 1 mM DTDA. The statistics of data collection, reduction and refinement are shown in Table 1. The general structure of the dimer was not altered by soaking with DTDA (Fig. 3, A) (Accession Number PDBI code 2OMA). The RMS deviation of the Cα traces of native TcTIM and the DTDA treated enzymes was 0.39 Å. The data also showed that in the position of Cys118, which is far from the interface, the two monomers exhibited electron densities that fitted well with a structure in which the sulfur of Cys118 was covalently linked through a disulfide bond to a thioaniline moiety (Fig. 3, A and B).Thus, in TcTIM crystals, DTDA was able to derivatize Cys118; however, as shown by the data on the Cys118Val mutant, the inhibition of activity by the compound does not depend on the perturbation of Cys118.

**Figure 4:**
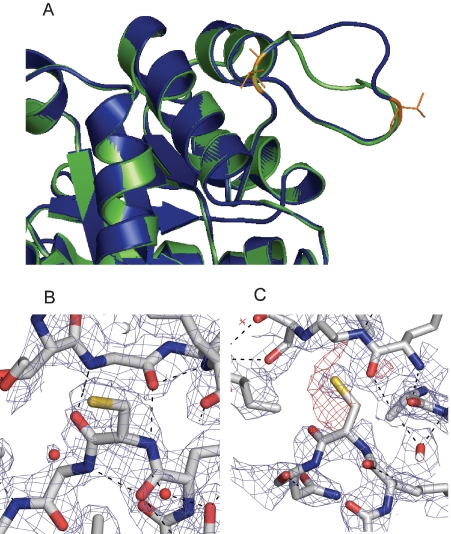
Structural alterations of the interfacial Cys15-loop3 regions of TcTIM induced by the soaking with DTDA. Panel A shows the superposition of loop3 of monomers A and B of the TcTIM crystal soaked with DTDA. It depicts the large scale difference in the conformations of loop3 of the “normal” monomer (B, in blue), and the “open” loop3 of monomer A (in green) in the span of residues 70–76. The hinge residues T70 and T76 are colored in orange. Panel B shows the interface region formed by Cys15 of monomer B and loop3 of the other monomer in native TcTIM. Panel C shows the same region in the DTDA treated enzyme. Please note that in native TcTIM, Cys15 is well defined, and that it establishes two H-bonds with Gly73 of the adjacent subunit (black dashed lines). These H-bonds do not exist in the DTDA treated enzyme. Electron density maps are included (2Fo-Fc, 1σ in blue and Fo-Fc, -3σ red), showing instability of Cys 15B.

Accordingly, and in regard to the inhibition of activity of TcTIM by DTDA, it is relevant that the enzyme soaked with DTDA exhibited significant and rather unique alterations in its dimer interface. One of the most notable was that although loop3 of monomer B exhibited a conformation almost identical to that in native TcTIM, loop3 of monomer A acquired a markedly different conformation (Fig, 4, A). Loop3 of TcTIM is formed by residues 66–79 (Q, N, A, I, T, R, S, G, A, F, T, G, E, and V). In monomer A of the DTDA treated enzyme, residues 66 to 70 and residues 77 to 79 superpose quite well with those of the “normal” loop, however, the region formed by residues 71 to 76 exhibited a significant displacement (Fig. 4, A) with a hinge at the level of Thr70 and Thr76. The different conformations that loops3 of monomers A and B adopted in the DTDA treated TcTIM are clearly evident in a superposition of the two (Fig. 4, A).

The change in conformation of loop3 of monomer A was accompanied by alterations in the contacts that it establishes with residues of monomer B. In *native* TcTIM, the side chain of Cys15 of the two subunits is well defined, and their N and O atoms are respectively, hydrogen bonded to the O and N atoms of Gly73 of the adjacent subunit (Fig. 4, B). In the DTDA treated enzyme, these H-bonds are missing (Fig. 4, C). This was consequence of an increase in the distance between Gly73 of monomer A and Cys15 of the other subunit; in the treated enzyme, the distances between N and O atoms of Gly73 and the O and N atoms of Cys15 were 7.9 and 9.2 Å, respectively. It is noted that the crystallographic data showed a negative electron density in the region of Cys15 of monomer B (Fig. 3, C). Taken together, the data indicate that the interactions of Cys15 of monomer B with Gly73 of the other subunit are less stable than in the wild type, and thus in all likelihood the inhibition of activity of TcTIM by DTDA is related to the alterations of the dimer interface. Along this line, it is also relevant that the B factors of residues 15 to 21 of monomer B in DTDA treated TcTIM had an average of 42.66 Å^2^, whereas in monomer A, the average values of the B factors of these same residues were 29.48 Å^2^. In native TcTIM, the average B factors of monomers A and B were 21.77 and 24.82 Å^2^, respectively.

### Effect of DTDA on *E. coli* that had TcTIM instead of their own TIM

**Figure 5:**
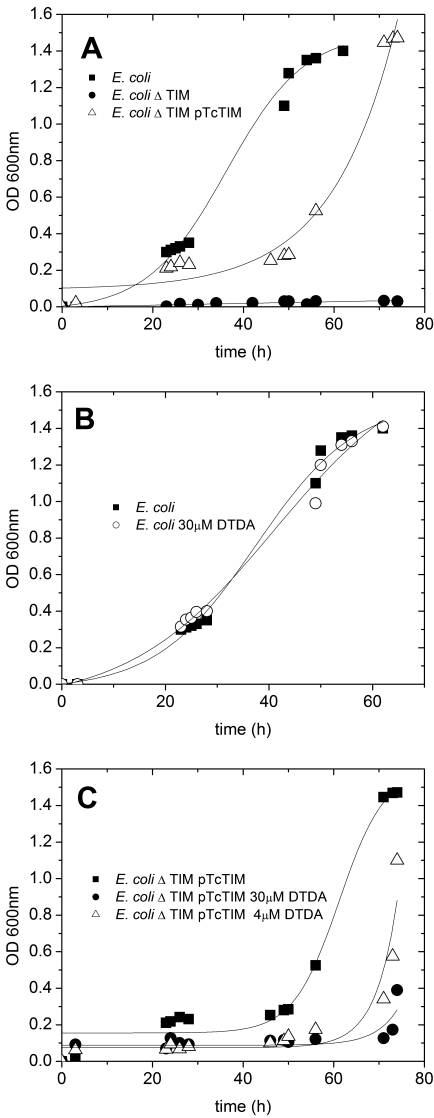
Growth of intact *E. coli*, of cells that lacked their endogeous TIM, and of *E. coli* cells in which their endogenous was replaced with TcTIM. Effect of DTDA. A shows the growth of intact *E. coli* (closed squares), *E. coli* whose TIM had been deleted (closed circles), and *E. coli* transformed with TcTIM (open triangles). Panel B depicts the growth curves of intact *E. coli* with and without 30 µM DTDA (closed squares and open circles, respectively). Panel C shows the growth curves of *E. coli* cells that had TcTIM instead of their own TIM with the indicated concentrations of DTDA.

In view of the powerful specific inhibiting effect of DTDA on TcTIM, we considered important to ascertain if the compound is able to cross biological membranes and whether it is detrimental to cells that rely on the presence of TcTIM. Accordingly, we determined the effect of the compound on *E. coli* cells that possessed their own TIM and in cells that depended on the function of TcTIM. These experiments involved three types of cells: i) Cells that have their endogenous TIM, ii) *E. coli* that lack their TIM, and iii) *E. coli* devoid of endogenous TIM that were transformed with TcTIM. Figure 5A shows the growth curve of intact cells in minimal media; the figure also shows that the growth of cells that are devoid of TIM is almost nil. These data therefore, illustrate that in minimal media, TIM is central to cell growth. In this respect it is particularly relevant that the growth of the latter cells was restored when they were transformed with TcTIM (Fig. 5. A), albeit the lag that precedes logarithmic growth was longer. Thus, the experiments show that the endogenous TIM of *E. coli* can be successfully replaced by the enzyme from *T. cruzi.*

When the effect of DTDA was assayed on the growth of normal and TcTIM transformed *E. coli*, it was found that 30 µM of the compound had no effect on cells that worked with their own TIM (Fig. 5, B). On the other hand, the growth of cells that relied on the function of TcTIM was effectively prevented by 30 µM DTDA (Fig. 5, C), and that concentrations as low as 4 µM induced an important increase in the lag that precedes logarithmic growth. Clearly, the data indicate that biological membranes are permeable to DTDA and that it can inhibit the activity of intracellular TcTIM.

### Effect of DTDA on cultured *T. cruzi* epimastigotes

**Figure 6:**
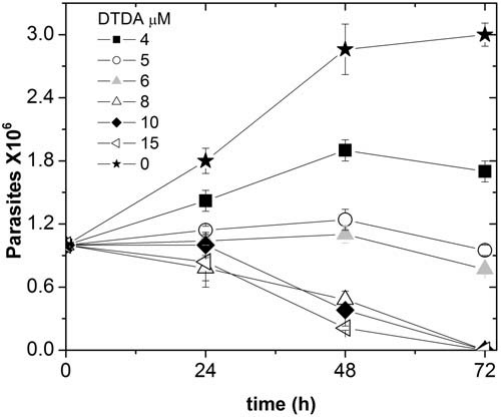
Effect of DTDA on *T.cruzi* epimastigotes. The parasites were cultured as described under Material and Methods with the indicated concentrations of DTDA.

The latter data prompted us to study the effect of DTDA on intact *T. cruzi.* To this end, 2 ml of RPM media that contained different concentrations of the compound were inoculated with 10^6^ *T. cruzi* epimastigotes, and incubated for 72 hours. The number of cells in the culture was determined every 24 hours. It was observed that at concentrations higher than 8 µM the compound brought about a significant decrease in the number of cells (Fig. 6). At lower concentrations (4 µM), DTDA brought about inhibition of growth. Thus, depending on its concentration, the compound, either prevented cell growth or caused the death of *T. cruzi* epimastigotes.

We would like to point out that there is a difference on the concentrations of DTDA that are effective on the pure enzyme and in whole cells. The former is inhibited by nM concentrations, whereas the adverse effects of the compound on TcTIM transfected *E. coli* and epimastigotes are observed with concentrations that are about 10 times higher. It is possible that *in vivo*, the binding of DTDA to the proteins that exist in the intracellular milieu, reduces its effective concentration. Although this phenomenon has been well documented for some pharmacological agents [34], at the moment it is not possible to offer a precise explanation for the difference in effectiveness of DTDA *in vitro* and in whole cells.

## Discussion

DTDA is a powerful inhibitor of the activity of TIM from *T. cruzi*. It is also effective in human TIM, but at concentrations that are nearly 400 times higher. Remarkably, the compound fails to affect the activity of TIM from *T. brucei*, and *L. mexicana*, albeit these enzymes are markedly similar to TcTIM in amino acid sequence and three-dimensional structure. An additional salient property of DTDA is that it is able to cross biological membranes as evidenced by the data with *E. coli*. These experiments also showed that DTDA does not affect the growth of intact *E. coli*, whereas in cells that depend on the function of TcTIM, low micromolar concentrations induce a strong inhibition of cell growth. These findings thus indicate that cell membranes are permeable to DTDA and that it affects adversely the life of cells that depend on the function of TcTIM. In consonance with these data, it was found that at concentrations of 4–8 µM, the compound induces a significant inhibition of the growth of *T. cruzi* epimastigotes, and that 10 µM and 15 µM causes death of parasites in cell cultures. Although the overall data suggest that the detrimental effect of DTDA on intact *T. cruzi* parasites is due to inhibition of the activity of their TIM, the results do not prove unambiguously that death of the parasites is due exclusively to the inhibition of that enzyme.

In regard to the properties of DTDA, it is relevant to point out that its effect and that of similar molecules on rodents have been previously reported. For example, it has been reported that the administration of 4,4′-diamino disulfide to rats induces hematological alterations [35], and that DTDA brings about irreversible oxidation of hemoglobin [36]; likewise, it was shown that diaminodiphenyldisulfide causes necrotic changes in the liver, and atrophy and hyperplasia in the kidney [37]. Although some disulfides, such as the ethanol deterrent disulfiram [38,39] have been successfully used for a long time as therapeutic agents, the observations on the toxicity of DTDA and similar molecules suggest that *per se*, this compound has no therapeutic value. However, the specific action of DTDA on TcTIM and the unique alterations that it induces in the structure of the enzyme suggest that it can be used as lead for the discovery of agents with pharmacological potential.

The experiments with pure recombinant wild type TcTIM and the mutants in which each of the four Cys was replaced with Val for the case of Cys118 or Ala in the other three Cys showed that regardless of their cysteine content, the four enzymes exhibit similar sensitivities to DTDA. Thus, it would appear that derivatization of cysteines is not the primary event in the inhibition of enzyme activity. On the other hand, the x-ray structure of native TcTIM that had been soaked in DTDA revealed that it induces important structural alterations of the dimer interface; the most obvious was on the interactions of loop3 of monomer A with the neighboring residues of the other subunit. Along this line, it is relevant to point out that because of its importance in catalysis and stability of the dimer, loop3 and its connections with the other subunit have been extensively studied. For example, it has been reported that perturbations of the interface region formed by Cys15 and loop 3 of the other monomer induce inhibition of activity [11–13]. In this connection it is relevant that Brown and Kollman [40] and Aqvist and Fothergill [41] showed by molecular modeling that during catalysis, the hydrogen bond of Thr76 of loop3 with the catalytic His96 of the other subunit shifts to the catalytic Glu. According to the authors, these arrangements are essential for the expression catalytic activity. Thus, it is noteworthy that the change in conformation of loop3 of monomer A had a hinge that localized to Thr76.

In connection to the contribution of loop3 to the stability of TIM dimers, it has been reported that deletions of some of the residues of loop3, yield enzymes that essentially exist in the monomeric form [9,10]. It is also relevant that the residues of loop3 surround the side chain of Cys15 of the other monomer, and that alkylation of the two interface Cys15 of *P. falciparum* [12] and *E. histolytica* [13] by thiol reagents induces the formation of stable monomers. In TIMs from *T. cruzi* and *T. brucei*, the derivatization of their two interface Cys15 induces aggregation of the enzymes [11]. In addition it is noteworthy that in a hybrid formed by a C15A TcTIM monomer and a monomer of wild type TbTIM, the alkylation of the only interface cysteine yields an enzyme that conserves its dimeric structure, albeit its catalytic properties are reduced by about one-half [42]. Therefore, it is mechanistically important that in the DTDA treated enzyme, loop3 of monomer A acquired a different position. This conformational change was accompanied by alterations of the contacts of Cys15 with the adjacent subunit; specifically, the electron density that corresponds to the β-carbon of Cys15 was not apparent, whereas that of the sulfur atom appeared diffuse. Moreover, DTDA treatment induced the loss of two H-bonds in the Cys15-loop3 interfacial region. The sum of these structural effects most likely accounts for its inhibiting effect of DTDA on the activity of TcTIM.

In the crystallographic data, there is another point that merits comment. This concerns the observation that even though in the TIM dimer, there are two equivalent Cys15-loop3 regions, only one of them was altered by DTDA, the other appeared intact. In solution the inhibiting effect of DTDA is accompanied by enzyme aggregation. This is in consonance with previous data [11] that showed that alterations of the dimer interface by chemical modification or site directed mutagenesis led to enzyme aggregation, indicating that perturbation of the interface lead to formation of unstable monomers that subsequently undergo aggregations. Therefore, it is likely that in the crystal, an intermediate of the overall conformational changes induced by DTDA was trapped.

In sum, this work shows that DTDA specifically inhibits the activity of TIM from *T. cruzi*, that it is able to cross biological membranes, and that it is effective in *T. cruzi* epimastigotes. An additional characteristic of DTDA is that it inhibits TcTIM by perturbing the interactions between its two subunits; thus, this compound is another example of the relatively small number of the so far reported agents that by acting on protein-protein interfaces induce a desired detrimental effect. Since the interfaces of oligomeric proteins would seem to be excellent targets for the discovery of agents that are specific for the enzymes from parasites [14,15], DTDA would seem to be a good model for the discovery of molecules that are less toxic, but that still conserve their effectiveness in the *T. cruzi* enzyme.
